# Low Tidal Volume Ventilation in Patients without Acute Respiratory Distress Syndrome: A Paradigm Shift in Mechanical Ventilation

**DOI:** 10.1155/2012/416862

**Published:** 2012-03-27

**Authors:** Jed Lipes, Azadeh Bojmehrani, Francois Lellouche

**Affiliations:** ^1^Institut Universitaire de Cardiologie et de Pneumologie de Quebec, Université Laval, Quebec, QC, Canada G1V 4G5; ^2^Department of Adult Critical Care, Jewish General Hospital, McGill University, Montreal, QC, Canada H3T 1E2; ^3^Centre de Recherche de l'Institut Universitaire de Cardiologie et de Pneumologie de Quebec, Université Laval, Quebec, QC, Canada G1V 4G5

## Abstract

Protective ventilation with low tidal volume has been shown to reduce morbidity and mortality in patients suffering from acute lung injury (ALI) and acute respiratory distress syndrome (ARDS). Low tidal volume ventilation is associated with particular clinical challenges and is therefore often underutilized as a therapeutic option in clinical practice. Despite some potential difficulties, data have been published examining the application of protective ventilation in patients without lung injury. We will briefly review the physiologic rationale for low tidal volume ventilation and explore the current evidence for protective ventilation in patients without lung injury. In addition, we will explore some of the potential reasons for its underuse and provide strategies to overcome some of the associated clinical challenges.

## 1. Introduction

Lung protective ventilation has evolved over the last several decades and has focused largely on patients suffering from acute respiratory distress syndrome (ARDS) and acute lung injury (ALI). There is clear evidence from animal and human data that mechanical ventilation can induce and exacerbate lung injury, and thus the current standard of care is the use of a lung protective ventilation strategy in patients suffering from ALI/ARDS [1–3]. Protective ventilation refers to the use of low tidal volume (VT), often in the range of 4–8 mL/kg of predicted body weight (PBW). In fact, 6 mL/kg is the normal physiologic VT in humans [4]. In ALI and in ARDS, there is a breakdown of normal lung architecture, loss of functioning lung units, and the development of high-permeability pulmonary edema, all of which result in clinically stiff, noncompliant, and heterogeneous lungs [5, 6]. The ventilator in this setting can produce a wide array of local and systemic adverse effects, known as ventilator-induced lung injury (VILI) [6, 7]. Mechanistically, these pathophysiologic changes occur from the direct effect of high pressure on the lung, barotrauma, from the damage caused by lung overdistension, volutrauma, from the shear stress of repetitive opening and closing of alveoli, atelectotrauma, and from the generation of cytokines and an inflammatory cascade, resulting in biotrauma. This can perpetuate lung injury as well as induce multiorgan failure, the most common cause of death in patients with ARDS [8–10]. Many investigators have conducted several large randomized trials that have shown that the use of lower VTs is associated with improved outcomes and a reduction in the incidence of VILI [11–15]. Indeed, over the last two decades, VTs in the intensive care unit (ICU) have been reduced, predominantly in patients with ALI or ARDS [16, 17]. However, evidence also exists that mechanical ventilation can be injurious to the lungs and other organ systems in patients without ALI or ARDS. We will review the current literature concerning the use of protective ventilation in non-lung injured patients and highlight important challenges that limit the successful implementation of protective ventilation in clinical practice.

## 2. The History of Protective Ventilation

In 1963, a seminal paper by Bendixen in the New England Journal of Medicine [18] demonstrated that the use of higher VTs during anesthesia (18 patients undergoing laparotomy) resulted in less atelectasis, less acidosis, and improved oxygenation compared to lower VTs. Nearly fifty years later, anesthesiology textbooks continue to maintain that VTs should be between 10–15 mL/kg while undergoing mechanical ventilation in order to avoid atelectasis and hypoxia [19]. Following the original description of ARDS by Ashbaugh et al. in 1967 [20], many studies have been performed in ALI/ARDS patients with a variety of VTs ranging from 10 to 24 mL/kg [21–24]. It was not until the 1970s and 1980s that a significant amount of data first started to emerge focusing on the deleterious effects of large VTs in the lungs of animals [7, 25, 26] and it was Hickling who first proposed the notion of permissive hypercapnia with the use of low VT [27, 28]. It was many years later when randomized clinical data emerged in humans revealing improved outcomes with the use of lower VTs with or without the use of high levels of positive end-expiratory pressure (PEEP), although initial data was conflicting [11–14, 29–31]. Amato et al. performed a randomized trial on 53 patients with ARDS, which revealed a reduction in 28-day mortality from 71% to 38% using 12 mL/kg versus 6 mL/kg [12]. In the same year, two other clinical trials emerged with conflicting results. A trial by Brochard et al. compared VTs of <10 mL/kg versus ≥10 mL/kg in 116 patients with ARDS and failed to show a significant reduction in mortality (37.9% versus 46.6%  *P* = 0.38), barotraumas, or multiorgan dysfunction [13]. Stewart et al. completed a similar trial in 120 patients comparing a VT of 8 mL/kg versus 10–15 mL/kg, which again did not result in a reduction in mortality (50% versus 47%, resp.) [14]. In 2006, Villar et al. randomized patients with ARDS to receive a VT of 9–11 mL/kg PBW with a PEEP ≥5 cm H_2_O or to a VT of 5–8 mL/kg PBW with a PEEP level setting based on the lower inflection point of the pressure-volume curve. They demonstrated an increase in ventilator free days and importantly a reduction in both ICU and hospital mortality [32].

In 2000, the largest and most influential trial was published by the Acute Respiratory Distress Syndrome Network, where they randomized 861 patients to a protective low VT strategy of 6 mL/kg of PBW with a plateau pressure less than 30 cm H_2_O, versus 12 mL/kg with a plateau pressure less than 50 cm H_2_O. They found a statistically significant reduction in mortality from 39.8% to 31% in the lower VT group [11]. Based on these results, current guidelines recommend VTs of 6 mL/kg of PBW for the management of patients with ALI or ARDS [33].

In addition to the reduction of tidal volume, increasing the level of PEEP is now considered an integral part of protective ventilation. The use of PEEP in ALI/ARDS and in particular at what level and how to set PEEP has not been without controversy. Coupled with a low tidal volume strategy three large randomized controlled trials have been published in the last decade: the ALVEOLI trial published in 2004 and the LOVS and EXPRESS trials in 2008 [29–31]. A recent meta-analysis, examining the data from the 3 trials which included 2299 individual patients, demonstrated that higher levels of PEEP were associated with improved survival among ARDS but not ALI patients [34].

The effect of low VT ventilation with higher levels of PEEP in patients without ALI or ARDS is less well known.

## 3. Protective Ventilation in Patients without Lung Injury

In addition to mechanical ventilation aggravating the course of disease in patients already suffering from ALI/ARDS, mechanical ventilation can also lead to the development of ALI/ARDS [35]. Once the lung has been “primed” by an initial physiologic insult such as pneumonia, aspiration, sepsis, blood transfusions or cardiopulmonary bypass, injurious mechanical ventilation can then initiate a pulmonary and systemic inflammatory response leading to ALI or ARDS [35]. Consequently, ventilated patients at highest risk for the development of further lung injury are those who are critically ill and undergo high-risk surgeries or who have an illness requiring an ICU admission (Tables 1 and 2). These patients may therefore benefit from a strategy of low tidal volume ventilation to reduce the risk of developing ALI/ARDS.

In 2004, Gajic et al. conducted a retrospective cohort study of mixed medical-surgical patients receiving mechanical ventilation for >48 hrs in the ICU who did not have ALI at the time of intubation [35]. This study analyzed 332 patients of which 80 (24%) developed ALI within 5 days of mechanical ventilation. Women tended to have higher VT (11.4 versus 10.4 mL/kg PBW) and developed ALI more frequently (29% versus 20%, *P* < 0.068). A multivariate analysis revealed an increased risk of developing ALI with the use of volumes greater than 6 mL/kg PBW (OR 1.3 for every 1 mL > 6 mL/kg, *P* < 0.001).

In another study by Gajic et al., analysis of a large international database of over three thousand mechanically ventilated medical-surgical ICU patients without ALI/ARDS showed that ARDS developed in 6.2% of patients after 48 hrs of mechanical ventilation. Most patients were admitted with sepsis, pneumonia, trauma or post-operatively. A multivariate analysis revealed that the use of a VT greater than 700 mL was associated with an odds ratio of 2.66 for the development of ARDS (*P* < 0.001) [36].

Determann et al. recently performed a randomized non-blinded two-centre trial comparing conventional 10 mL/kg PBW to 6 mL/kg PBW in 152 critically ill medical/surgical patients [37]. Patients were randomized within 36 hours of initiation of mechanical ventilation. The primary outcome was the measurement of cytokine levels in bronchoalveolar lavage (BAL) fluid and plasma. Secondary outcomes included the development of ALI/ARDS, duration of ventilation, and overall mortality. Despite balanced baseline demographics including known risk factors for ALI/ARDS, the trial was stopped early when more patients developed lung injury in the conventional group, 13.5% versus 2.6% (*P* = 0.01). The relative risk for developing lung injury with conventional ventilation was 5.1 (95% CI, 1.2–22.6). Plasma IL-6 levels decreased over 4 days and were more pronounced in the low VT group (21 ng/mL versus 11 ng/mL, *P* = 0.01). BAL cytokine levels including IL-6 tended to be higher in the conventional group, although it was not statistically significant.

A multicenter randomized controlled trial by Mascia et al. enrolled 118 potential organ donors with normal lungs, who received either conventional or protective ventilation [38]. Current guidelines for the management of organ donors suggest 8–15 mL/kg of VT [39, 40]. Patients receiving conventional ventilation were treated with 10–12 mL/kg PBW with a PEEP of 3–5 cm H_2_0 for six hours, whereas patients receiving protective ventilation were treated with 6–8 mL/kg PBW with a PEEP of 8–10 cm H_2_0 for the same length of time. The results revealed a dramatic increase in the number of eligible lung donors (54% versus 95%, *P* < 0.001), as well as in the number of patients in whom lungs were harvested (27% versus 54%, *P* = 0.004), suggesting either a decrease in lung damage due to a less exposure to injurious standard VT ventilation or better lung recruitment with higher levels of PEEP.

The data for high-risk surgical patients also appears to favor the use of prophylactic lung protective ventilation. A trial by Lee et al. in a surgical ICU, excluding neurotrauma and cardiac surgery patients, randomized 103 patients to 12 mL/kg versus 6 mL/kg. They documented a reduction in pulmonary infections, a trend towards a reduced ICU length of stay and a reduced duration of intubation in the group with lower VTs [41]. Fernández-Pérez et al. analyzed, via retrospective chart review, 170 patients undergoing pneumonectomy. The analysis showed that of all the patients 18% developed respiratory failure and 50% of these developed ALI. Those who developed respiratory failure had higher intraoperative VTs compared to those who did not (8.3 versus 6.7 mL/kg *P* < 0.001, OR 1.56) [42]. Michelet et al. performed a randomized trial with 52 cancer patients undergoing esophagectomy, comparing a VT of 9 mL/kg and zero PEEP (ZEEP) versus 9 mL/kg during two-lung ventilation which was reduced to 5 mL/kg during one-lung ventilation with a PEEP of 5 cm H_2_0 [43]. Postoperatively, a reduction in inflammatory cytokines and, more importantly, improved PaO_2_/FiO_2_ ratios, and a reduction in the duration of intubation were found in the reduced VT group. A small randomized trial of 40 elderly patients undergoing major abdominal surgery compared a VT of 10 mL/kg and ZEEP with 6 mL/kg and 12 cm H_2_0 of PEEP with recruitment maneuvers. The major finding was that an “open lung” protective ventilation strategy was tolerated hemodynamically, and despite no change in inflammatory biomarkers, the low VT-high PEEP group had improved intraoperative oxygenation and respiratory mechanics [44].

Conflicting data does exist for high-risk surgical patients. A large prospective case control trial of 4420 consecutive patients undergoing major surgery lasting for >3 hours examined intraoperative ventilator settings and the incidence of ALI, and survival. Three percent of patients (*n* = 83) developed ALI and it was found that the mean airway pressure in the first hour of ventilation correlated with the development of ALI but no correlation was demonstrated between VT or PEEP and the development of ALI [45]. In addition, in a recent retrospective study of 89 surgical patients admitted with respiratory failure, Hughes et al. were unable to find any correlation between intraoperative VT and the development of ARDS, although most patients in this study were being ventilated with a VT of <10 mL/kg PBW [46].

Cardiac surgery patients seem to be at higher risk of acquiring ALI/ARDS due to the inflammatory changes caused by cardiopulmonary bypass [47, 48]. In cardiac surgery, a number of studies have been performed examining clinical and biochemical changes associated with varying VTs. A reduction in plasma and bronchoalveolar cytokine levels was seen when using a low VT strategy (8 mL/kg and PEEP of 10 compared to 10–12 mL/kg and PEEP of 2-3 cm H_2_O) in 40 randomized elective coronary artery bypass surgeries [49]. Other small trials revealed similar reductions in inflammatory markers, although with conflicting results [50–52]. Sundar et al. randomized elective cardiac surgery patients to receive a VT of either 6 or 10 mL/kg PBW. Patients receiving lower VTs were more likely to be liberated from ventilation at 6 hrs after surgery. It is not clear if these effects were related to a strategy of protective ventilation or if they were due to an increase in respiratory drive by the effect of low VT inducing higher PaCO_2_ and lower pH levels. Moreover, fewer patients with low VT required reintubation [53].

The largest study concerning cardiac surgery patients examined 3434 consecutive bypass, valve or combined procedures, and the immediate postoperative VTs used. VTs were divided into low (<10 mL/kg PBW), traditional (10–12 mL/kg PBW), or high (>12 mL/kg PBW). A multivariate analysis comparing high versus low VT revealed an increased risk of mechanical ventilation lasting more than 24 hrs (OR 2.4, *P* = 0.001), hemodynamic instability for more than 48 hrs (OR 1.8, *P* = 0.007), and a prolonged ICU stay for more than 7 days (OR 1.8, *P* = 0.045) in the higher VT group. In addition, risk factors for the use of high VT included a BMI > 30 (OR 6.2, *P* < 0.0001) and female sex (OR 4.3, *P* < 0.0001) [54].

The data for the use of low VT in patients undergoing lower-risk elective operations is less evident. A trial of 39 low-risk patients undergoing elective surgery randomized patients to 15 mL/kg PBW with ZEEP, 6 mL/kg PBW with ZEEP, or 6 mL/kg PBW with a PEEP of 10 cm H_2_0. Plasma cytokine levels were measured after 1 hour of mechanical ventilation. The authors were unable to find any difference in inflammatory biomarkers in any of the groups [55]. In another small trial, investigators randomized patients undergoing elective thoracotomy to receive a VT of 12 mL/kg PBW with ZEEP versus 6 mL/kg PBW with 10 cm H_2_O of PEEP and also randomized patients undergoing elective laparotomy to a VT of 15 mL/kg PBW with ZEEP or 6 mL/kg PBW with 10 cm H_2_O of PEEP. After 3 hours of ventilation, inflammatory mediators including TNF, IL-1, 6, 8, 10, and 12 were measured in both the plasma and tracheal secretions. They too were unable to find a significant difference between the two groups [26]. Whether cytokine levels are a meaningful endpoint or whether or not 3 hours is sufficient to find a difference may both be limiting factors. Choi et al. analyzed pulmonary inflammation in the bronchoalveolar fluid of 40 patients undergoing elective complex abdominal surgeries with a minimum duration of 5 hrs. Patients were randomized to 12 mL/kg PBW with ZEEP or 6 mL/kg PBW with 10 cm H_2_0 of PEEP. Although no clinical differences were found between groups (the study was not powered to find clinical differences), they did find a significant increase in procoagulant biomarkers in patients ventilated with elevated VTs [56].

## 4. The Clinical Application of Lung Protective Ventilation

The overall goal of lung protective ventilation is to minimize lung trauma by avoiding both overdistention (and associated elevated pressure) and repetitive alveolar collapse, while providing adequate oxygenation and ventilation. What is important to note is that the peak pressure is not necessarily the distending pressure transmitted to the alveoli, and consequently elevated peak pressures are not detrimental to the lung per se. The actual distending pressure of the alveoli is better reflected by the plateau pressure, which can be easily achieved by performing an inspiratory pause on the ventilator in a volume-controlled mode. In truth, the transmitted pressure or transpulmonary pressure, calculated as the difference between the alveolar and pleural pressures [57], is what causes alveolar trauma, although it can be more difficult to measure and monitor at the bedside. As such, to minimize the risk of developing VILI, one should monitor and maintain the plateau pressure below 30 cm H_2_0, unless your ICU has the capability of reliably measuring transpulmonary pressure.

To avoid alveolar overdistention and volutrauma, a VT of 6 mL/kg PBW is recommended for ALI or ARDS patients. Of note, it is crucial that the PBW and not the actual body weight (ABW) be used to calculate tidal volume. The use of ABW may overestimate the required VT [58]. In patients without ALI/ARDS current evidence suggests that a VT between 6–8 mL/Kg PBW in patients at risk of ALI/ARDS should be used, and, in patients without risk factors, a VT ≤ 10 mL/kg PBW is appropriate.

PEEP has several benefits in lung protective ventilation. PEEP is used to keep the alveoli open and to minimize atelectotrauma: a “lung-open” strategy. PEEP also protects from alveolar derecruitment and secondary hypoxemia while minimizing the amount of inspired oxygen required, thereby avoiding denitrogenation atelectasis and potential oxygen toxicity [59]. Another way to avoid atelectasis is to reduce the FiO_2_, as levels above 60% can cause denitrogenation atelectasis [60–62]. To this end, the SpO_2_ target must be reduced in the case of ARDS patients to 88–92% [33, 63]. From clinical data, PEEP ranging between 5 and 24 cm H_2_0 is not only safe but is associated with improved oxygenation, less need of rescue therapy for refractory hypoxemia, and reduced multiorgan failure [29, 30, 34]. Although the optimal level of PEEP is still controversial, the use of zero PEEP (ZEEP) has been associated with worse outcomes, including increased hypoxemia, ventilator-associated pneumonia, and hospital mortality [64, 65]. How and to what level PEEP should be titrated to remains unclear. Some authors have used pressure-volume curves and the lower inflection point to titrate PEEP with good results [32]. The ALVEOLI and LOVS trials used a set PEEP sliding scale based on the FiO_2_ with a maximum plateau pressure of 30 or 40 cmH_2_0, respectively [29, 30]. The EXPRESS trial used the maximum amount of PEEP as long as the peak pressure remained below 30 cm H_2_0 [31]. In a pilot study by Talmor et al. patients with ARDS were randomized to a strategy of esophageal balloon directed estimation of pleural pressures and consequently used the calculated transpulmonary pressures to guide PEEP titration. The control group was managed as per the ARDS Network protocol including a PEEP sliding scale based on FiO_2_. This single centre pilot trial demonstrated a significant improvement in oxygenation, pulmonary compliance, and 28-day mortality [66]. In patients without ALI/ARDS, PEEP levels between 5 and 12 cm H_2_O have been used in conjunction with low tidal volume and usually ≥8 cm H_2_O (Tables 1 and 2). Therefore we suggest starting with a PEEP level of 8 cm H_2_O and titrating up or down depending on the FiO_2_ and the hemodynamic status of the patient.

Due to the reduction in VT and subsequent minute ventilation, carbon dioxide levels are often elevated in these patients and the hypercapnia tolerated, a concept referred to as permissive hypercapnia [67]. To avoid severe respiratory acidosis (ph < 7.20), the respiratory rate needs to be increased, often up to 30 breaths/min. For patients with risk factors for ARDS such as sepsis, a starting respiratory rate of 20 or more is suggested to avoid severe acidosis following initial intubation and ventilation with 6–8 mL/kg PBW. In addition, the humidification system used with the ventilator is particularly important and can be a significant contributor to increased respiratory acidosis. To reduce the severity of hypercapnia, reduction of dead space can be easily accomplished by using a heated humidifier instead of a heat and moisture exchanger [68–74] (Table 3).

## 5. Challenges and Controversy to the Use of Protective Ventilation

Although convincing data has accumulated in the literature, protective ventilation is not as widely used as it should be, and there remains some controversy in the most appropriate way to prescribe and titrate lung protective ventilation [75]. Should all patients with ARDS receive the same 6 mL/kg PBW of ventilation? This question has been raised by a number of leading authors in the field of mechanical ventilation and ARDS and remains controversial [76–78]. Although the results of the ARMA trial were positive, other similar smaller trials were not [11–15], and there is evidence that the correlation between PBW, height, and lung volume is lost in patients with ARDS as compared to normal controls [79, 80]. Furthermore, the lung strain and potential injury induced by a given VT is dependant on the amount of “baby lung,” and this is highly variable between patients [81, 82]. In an editorial in 2005, Deans et al. highlight the relationship between pulmonary compliance and mortality in the ARMA trial. Patients with a low compliance had a higher mortality if receiving a higher VT. However, patients with higher baseline pulmonary compliance had an increase in mortality when receiving a lower VT therefore suggesting that all patients should not receive the same prescribed VT [76]. Gattinoni suggests a tailored approach to setting up mechanical ventilation with measurements of lung volume and transpulmonary pressures, although her admits that, should these methods not be possible in an individual centre, a lower VT compared to a higher VT is almost always more appropriate [77].

 There are multiple reasons why clinicians underuse low-VT ventilation. There is a general underrecognition of ALI and ARDS in clinical practice, and, consequently, many patients who could benefit from this therapy are not receiving appropriate treatment [75, 83]. In an attempt to identify patients less likely to receive lung protective ventilation, a recent study found that demographic factors including height, race, and lower simplified acute physiology II (SAPS II) scores were associated with underuse of low-VT ventilation [84].

The use of protective ventilation does require more advanced knowledge of ventilator management, and, depending on the availability of local expertise, clinicians may be unable or reluctant to make the necessary adjustments. In order to maintain adequate minute ventilation, one is often required to aggressively increase the respiratory rate, sometimes above 30 breaths/min. This may lead to the development of dynamic hyperinflation and auto-PEEP which can have significant negative respiratory and hemodynamic consequences [85]. Due to short time constants and low lung compliance in patients with ALI/ARDS, this risk is usually limited below 30 breaths/minute [86]. However, the ability to recognize patients at risk for auto-PEEP as well as to accurately recognize the presence of dynamic hyperinflation from abnormal ventilator waveforms is fundamental to safe clinical practice [87]. Quickly analyzing the flow and pressure waveforms of most ventilators can identify most patients with dynamic hyperinflation or auto-PEEP. When the end-expiratory flow is below the zero baseline, dynamic hyperinflation exists. By performing an expiratory flow occlusion maneuver, the static auto-PEEP can be measured which corresponds to the mean PEEP of all lung units [88].

An additional concern associated with low tidal volume ventilation is the development of significant patient-ventilator dyssynchrony. Dyssynchrony can occur in any patient who is mechanically ventilated and in particular patients who are not sedated or who have a high drive to breathe. Although many types of patient-ventilator dyssynchrony occur, the most important for ARDS patients relates to flow dyssynchrony, which occurs when the volume or flow delivered to the patient fails to meet the respiratory demand or the neural drive to breathe [89]. Dyssynchrony cannot only cause intense patient anxiety and a sense of dyspnea, which occurs commonly in ventilated patients, but can increase the work of breathing and cause respiratory muscle fatigue [90–92]. In order to minimize patient-ventilator dyssynchrony, the clinician must routinely examine the patient for signs of distress and the ventilator for abnormal waveforms [93]. Titrating the inspiratory flow based on both the physical exam and waveform analysis can help minimize the dyssynchrony and usually requires a flow of at least 80 L/min but should be individualized for each patient and titrated when needed. The use of a decelerating flow waveform can also be used to deliver high inspiratory flows with a relatively longer inspiratory time which can avoid the problem of double triggering that occurs with the shorter inspiratory times associated with a square waveform [92]. Some patients with high minute ventilation remain dyssynchronous with tidal volumes of 6 mL/kg PBW and either require heavy sedation, paralysis, or an increase in VT. However, in centers experienced with ALI/ARDS, a low tidal volume strategy did not overall increase the use of sedation, and the recent large randomized trial examining the use of neuromuscular blockers in acute ARDS has alleviated some concerns regarding the risks of sedation and paralysis if needed [94, 95]. Moreover, in the initial ARMA study, patients were allowed an increase in VT to 8 mL/kg PBW if significant dyssynchrony occurred, as long as the plateau pressures remained below 30 cm H_2_O, and in a study by Kallet et al., a tidal volume of 8 mL/kg PBW was associated with a reduction in the work of breathing; therefore this may be an appropriate maneuver to avoid severe patient-ventilator dyssynchrony [91].

Physicians' additional concerns for hypercapnia, acidosis, and hypoxemia have also led to barriers in implementing protective ventilation [96]. Permissive hypercapnia should be tolerated in patients undergoing protective ventilation although to what degree is still unclear. In an attempt to determine the safety and lung protective effects of extreme hypercapnia, surfactant depleted rabbits were ventilated with progressively lower VTs and targeted a progressively higher PaCO_2_. There was significantly less lung injury when ventilated with a VT of 4-5 mL/kg and an associated PaCO_2_ of 80 mmHg (pH = 7.09) compared to 8–10 mL/kg with a PaCO_2_ of 40 mmHg (pH = 7.29). Even in animals with a VT of 3-4 mL/kg and a PaCO_2_ of 120 mmHg (pH = 6.98) or 2-3 mL/kg and a PaCO_2_ of 160 mmHg (pH = 6.91), lung protection was preserved and without hemodynamic consequences [97]. Knowledge of the potential adverse effects of hypercapnia on the cardiovascular, respiratory, and other organ systems is still critical in order to avoid serious adverse effects. Some important physiologic considerations due to hypercarbia include the negative effects of acidosis on cardiovascular hemodynamics, pulmonary hypertension due to pulmonary arterial vasoconstriction, and elevation of intracranial pressure due to cerebral vasodilation [67]. In our practice we tolerate respiratory acidosis with pH values as low as 7.20 in very severe ARDS patients after dead space minimization and optimization of the respiratory rate have been performed, but the lower acceptable value is not clearly determined.

Hypoxia secondary to derecruitment atelectasis associated with low VT ventilation, although a concern, should be placed in the appropriate context. It is recommended to target a SpO_2_ of 88–92% in ARDS patients in order to minimize the delivered FiO_2_, and the use of both recruitment maneuvers and higher levels of PEEP in nonhypovolemic patients may be helpful to reduce FiO_2_ requirements in certain clinical situations (following ventilator disconnect for suctioning or transport) but should not be systematically used. In the landmark ARDS Network study, the reduction in VT was associated with a lower PaO_2_, which did not impede the reduction in overall mortality [11]. Indeed, clinicians often target oxygen levels that are excessively high. A recent analysis examined clinicians' response to hyperoxia in 5498 mechanically ventilated patients. In this study, 22% of arterial blood gases had PaO_2_ levels greater than 120 mmHg, and in only 25% of those blood gases, the FiO_2_ levels were adjusted [98]. Studies examining the effects of hyperoxia in mechanically ventilated animal lungs have shown that high FiO_2_ is associated with increased oxidative injury, impaired immunity, and death [99]. Large clinical trials in humans are unfortunately lacking. However, human data examining the physiologic effects of hyperoxia does exist and includes impaired myocardial blow flow, increased myocardial consumption, and reduced cerebral blood flow due to arterial vasoconstriction [100–102]. Thus, in patients ventilated without lung injury, where hyperoxia is easily obtained, one should minimize FiO_2_ levels to avoid potential systemic oxygen toxicity.

It takes many years to implement research findings into clinical practice, a process referred to as knowledge translation [103, 104]. Indeed, despite the evidence showing that a reduced VT strategy is associated with improved outcomes, clinicians still routinely use VTs greater than 10 mL/kg [105, 106]. Eight years following the original landmark ARDS Network paper, Umoh et al. still found that only 46% of eligible patients received low VT ventilation in nine ICUs of three different teaching hospitals [106]. Studies showing the best implementation of protective ventilation were carried out in centers participating in mechanical ventilation networks [107], but this does not reflect real-life practice, where protective ventilation in ARDS patients is even less successfully applied [108].

**Figure 1:**
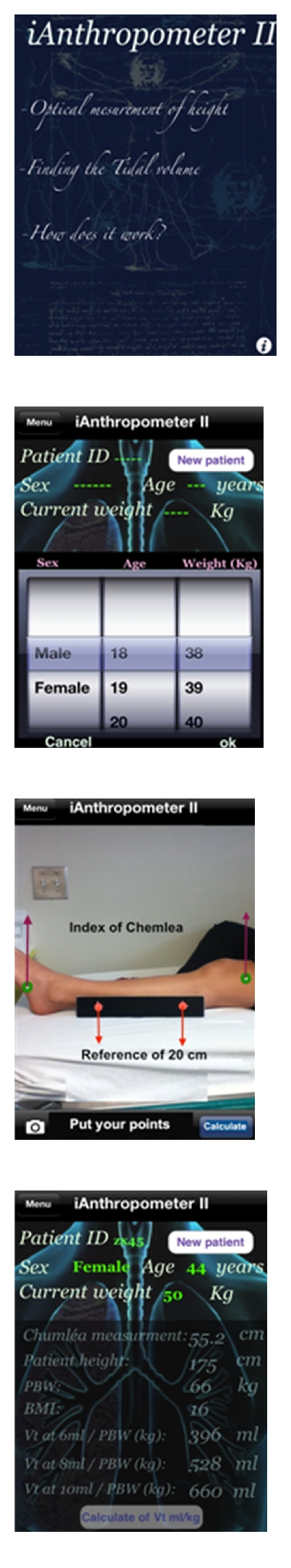
iAnthropometer II smartphone application for the assessment of PBW [113]. This smartphone application facilitates the calculation of patient height via digital measurement of leg length and subsequently calculates the PBW and the appropriate corresponding VT.

Even when ICU physicians attempt to use protective ventilation in their everyday practice, the use of actual instead of PBW in the calculation of VT is a frequent error, leading to overtreatment with higher VTs. PBW in men is calculated as 50 (45 in women) + 0.91 (Height cm −152.4) [11]. In many instances, the height of the patient is not immediately known. This is especially true outside of the surgical ICU (i.e., emergency department, postanesthesia recovery room or medical ICU) because surgical ICUs are more likely to have height and weight measurements due to the operative record [58]. In addition, visual estimation of patient height and weight is known to be inaccurate [109–111], and shorter patients, often women, tend to be more severely affected [54, 112]. The ability to rapidly calculate PBW at the bedside is important. Novel devices such as applications on smartphones, (e.g., iAnthropometer ICU), where one can quickly take a bedside picture of a patients leg and calculate the patients height based on validated formulas, and automatically derive the PBW and subsequent VTs, are promising tools (Figure 1) [113]. This application was more accurate at calculating patient height than both the method of visual estimation and supine in-bed tape measurement [114].

Automated mechanical ventilation systems are another way to help implement protective ventilation. In a preliminary study, we evaluated the new Intellivent system (Hamilton medical, Bonaduz, Switzerland) in patients after cardiac surgery [115]. We showed that VTs were automatically reduced below 10 mL/kg of PBW after a few minutes of mechanical ventilation, and the respiratory rate was automatically increased to maintain a stable minute ventilation. These closed-loop systems will likely become more widely available given the results of promising initial clinical evaluations [116–119].

## 6. Conclusion

The routine use of protective ventilation in all ventilated patients is not yet recommended, but more liberal use of this currently restricted treatment may be justified. Schultz et al. recommend a VT of 6 mL/kg PBW in patients with ALI/ARDS or in patients with risk factors for the development of lung injury [120], such as multiple transfusions, trauma, sepsis, or high-risk surgery. In other mechanically ventilated patients, they recommend the use of VTs below 10 mL/Kg PBW from the initiation of mechanical ventilation. This is in agreement with our findings in patients undergoing cardiac surgery [54]. Whether there is a benefit in other subgroups of patients, such as those intubated for congestive heart failure or other high-risk surgeries or trauma patients, is unknown, and further studies are needed. Moreover, it remains unclear if and how VT should be individualized. As technology improves, measurements of lung volume and transpulmonary pressures may become more technically feasible, affordable, and reliable. However for the majority of clinicians this practice is currently restricted to selected specialized centers and to the field of research.

Given the unpredictability of developing ALI/ARDS throughout the course of apost illness, a ventilation strategy that incorporates low VT and high PEEP in the majority of intubated patients is warranted and is in agreement with other authors [78, 104]. This paradigm shift in the management of mechanical ventilation changes the goal of treatment from limiting disease progression (i.e., in patients already suffering from ALI/ARDS) to disease prevention. Patients who may not benefit from this strategy are those who are otherwise healthy and are undergoing routine elective surgery. Given the proven safety of this approach, the physiologic rationale, and the current evidence, mechanically ventilated patients who do not have ARDS but who remain at risk for ALI/ARDS should be ventilated with a VT between 6–8 mL/kg PBW while those without risk factors should receive ≤10 mL/kg PBW to prevent progressive lung injury. Compared to patients with ARDS who often have a significant amount of recruitable lung units and higher oxygenation requirements, the amount of PEEP required for patients without ARDS is therefore much less, and based on current evidence, we suggest an initial setting of 8 cm H_2_O which should be titrated based on the individual patients oxygenation requirements.

There is clear evidence from large randomized trials that protective ventilation can reduce morbidity and mortality in patients with ALI and ARDS. More recently, significant data has emerged which suggests that a protective ventilation strategy in patients without ALI/ARDS may lead to a reduction in inflammation, less organ dysfunction, and less ALI. Patients at highest risk are those within the ICU with sepsis, trauma, or shock, high-risk surgical patients, or those undergoing cardiopulmonary bypass. How this therapy should be individualized to patients with variable respiratory and cardiovascular physiology remains controversial and future studies will be required to identify if all patients undergoing mechanical ventilation would benefit from lower tidal volumes, lower plateau pressures, and higher levels of PEEP.

## Figures and Tables

**Table 1:** Impact of ventilation strategy after noncardiac and cardiac surgery.

Reference (year)	Type of patients (*n*)	VT-PEEP	Main results
		mL/kg cm H_2_O	
Non-cardiac surgery			
Fernández-Pérez et al. (2006) [42] Observational	Pneumonectomy (170)	VT 8.3 versus 6.7	18% postoperative ARF VT was a risk factor for ARF
Michelet et al. (2006) [43] RCT	Esophagectomy (52)	VT 9/9-PEEP 0 VT 9/5-PEEP 5	*↘* inflammatory markers *↗* oxygenation, *↘* MV duration
Lee et al. (1990) [41] RCT	Mixed postop patients (103)	VT 12 versus 6	Trend for *↘* pulmonary infections and *↘* MV duration
Wrigge et al. (2000) [55] RCT	Elective surgeries (39)	VT 15-PEEP 0 VT 6-PEEP 0 VT 6-PEEP 10	No difference in inflammatory markers 1 h after surgery
Wrigge et al. (2004) [26]RCT	Abdominal and thoracic surgeries (64)	VT 12–15-PEEP 0 VT 6-PEEP 10	No difference in inflammatory markers 3 hrs after surgery
Choi et al. (2006) [56] RCT	Prolonged surgeries (40)	VT 12-PEEP 0 VT 6-PEEP 10	*↘* coagulation activation after 5 hrs of MV
Weingarten et al. (2010) [44] RCT	Major abdominal surgeries (40)	VT 10-PEEP 0 VT 6-PEEP 12	Improved respiratory mechanics and oxygenation, no difference in biomarkers

Cardiac surgery			
Koner et al. (2004) [51] RCT	CABG (44)	VT 10-PEEP 0 VT 6-PEEP 5	No difference on inflammation *↗* oxygenation with PEEP
Wrigge et al. (2005) [52] RCT	CABG (44)	VT 6-PEEP 10 VT 12-PEEP 7 VT 6-PEEP 9	*↘* TNF in BAL
Reis Miranda et al. (2005) [50] RCT	CABG (62)	VT 6–8-PEEP 5 VT 4–6-PEEP 10	More rapid *↘* in proinflammatory cytokines
Zupancich et al. (2005) [49] RCT	CABG (40)	VT 10–12*-PEEP 2-3 VT 8*-PEEP 10	*↘* proinflammatory cytokines after cardiopulmonary bypass
Sundar et al. (2011) [53] RCT	CABG, Valves (149)	VT 10-PEEP > 5 VT 6-PEEP > 5	Less intubated patients after 6 hrs Less reintubation
Lellouche et al. (2010) [54] Observational	CABG, Valves (3434)	VT < 10 versus 10–12 versus > 12	*↗* organ dysfunction and *↗* ICU length of stay with high and traditional VT

RCT: randomized controlled studies. MV: mechanical ventilation. ARF: acute respiratory failure. BAL: bronchoalveolar lavage. TNF: tumour necrosis factor. VT: tidal volume.

*mL/Kg of actual body weight (ABW).

**Table 2:** Impact of ventilation strategy for critically ill intensive care unit patients.

Reference (year)	Type of patients (*n*)	VT-PEEP	Main results
		mL/kg cm H_2_O	
Intensive care unit patients			
Gajic et al. (2004) [35] Cohort	Medical/surgical (332)		OR for ALI = 1.3 for every 1 mL > 6 mL/kg

Gajic et al. (2005) [36] Retrospective	Medical/surgical (3261)		OR for ARDS = 2.6 with VT > 700 mL

Determann et al. (2010) [37] RCT	Medical/surgical (150)	VT 10 versus 6	Relative risk for developing ALI was 5.1 with high VT ↓ inflammatory markers

Mascia et al. (2010) [38] RCT	Organ Donors (118)	VT 10–12-PEEP 3–5 VT 6–8-PEEP 8–10	More eligible and harvested lungs with low VT

RCT: randomized controlled studies. MV: mechanical ventilation. VT: tidal volume. ALI: acute lung injury. ARDS: acute respiratory distress syndrome.

**Table 3:** Recommended initial lung protective mechanical ventilator settings following intubation in patients without ALI/ARDS.

	Patients without risk factors for ALI/ARDS	Patients with risk factors for ALI/ARDS*
VT (mL/kg PBW)	<10	6–8
Respiratory rate (breath/min)	≥15	≥20
PEEP (cm H_2_O)	≥5	≥8
FiO_2_ (%)	<60**	<60**
Target SpO_2_ (%)^†^	92–96	92–96
Humidification device	HME***	HME***

*Major risk factors for acquired ALI/ARDS include: Sepsis, trauma, blood transfusions, cardiopulmonary bypass, and others.

**The lowest FiO_2_ to achieve an acceptable SpO_2_ should be used.

***The heterogeneity of the HME should be known, and, if severe respiratory acidosis occurs, heated humidifiers should be used instead [68].

^†^If FiO_2_ requirements are above 60%, a target SpO_2_ of 88–92% should be tolerated [63].

VT: tidal volume, PBW: predicted body weight, and HME: heat and moisture exchangers.

## References

[1] Webb HH, Tierney DF (1974). Experimental pulmonary edema due to intermittent positive pressure ventilation with high inflation pressures. Protection by positive end expiratory pressure. *American Review of Respiratory Disease*.

[2] Dreyfuss D, Soler P, Basset G, Saumon G (1988). High inflation pressure pulmonary edema. Respective effects of high airway pressure, high tidal volume, and positive end-expiratory pressure. *American Review of Respiratory Disease*.

[3] Slutsky AS (2001). Basic science in ventilator-induced lung injury: implications for the bedside. *American Journal of Respiratory and Critical Care Medicine*.

[4] Tenney SM, Remmers JE (1963). Comparative quantitative morphology of the mammalian lung: diffusing area. *Nature*.

[5] Ware LB, Matthay MA (2000). The acute respiratory distress syndrome. *New England Journal of Medicine*.

[6] Granton JT, Slutsky AS, Hall J, Schmidt G, Wood L (2005). Ventilator-induced lung injury. *Principles of Critical Care*.

[7] Dreyfuss D, Saumon G (1998). Ventilator-induced lung injury: lessons from experimental studies. *American Journal of Respiratory and Critical Care Medicine*.

[8] dos Santos CC, Slutsky AS (2004). Protective ventilation of patients with acute respiratory distress syndrome. *Critical Care*.

[9] Tremblay LN, Slutsky AS (1998). Ventilator-induced injury: from barotrauma to biotrauma. *Proceedings of the Association of American Physicians*.

[10] Plötz FB, Slutsky AS, Van Vught AJ, Heijnen CJ (2004). Ventilator-induced lung injury and multiple system organ failure: a critical review of facts and hypotheses. *Intensive Care Medicine*.

[11] Brower RG, Matthay MA, Morris A, Schoenfeld D, Thompson BT, Wheeler A (2000). Ventilation with lower tidal volumes as compared with traditional tidal volumes for acute lung injury and the acute respiratory distress syndrome. *New England Journal of Medicine*.

[12] Amato MBP, Barbas CSV, Medeiros DM (1998). Effect of a protective-ventilation strategy on mortality in the acute respiratory distress syndrome. *New England Journal of Medicine*.

[13] Brochard L, Roudot-Thoraval F, Roupie E (1998). Tidal volume reduction for prevention of ventilator-induced lung injury in acute respiratory distress syndrome. *American Journal of Respiratory and Critical Care Medicine*.

[14] Stewart TE, Meade MO, Cook DJ (1998). Evaluation of a ventilation strategy to prevent barotrauma in patients at high risk for acute respiratory distress syndrome. *New England Journal of Medicine*.

[15] Eichacker PQ, Gerstenberger EP, Banks SM, Cui X, Natanson C (2002). Meta-analysis of acute lung injury and acute respiratory distress syndrome trials testing low tidal volumes. *American Journal of Respiratory and Critical Care Medicine*.

[16] Esteban A, Anzueto A, Frutos F (2002). Characteristics and outcomes in adult patients receiving mechanical ventilation: a 28-day international study. *Journal of the American Medical Association*.

[17] Wongsurakiat P, Pierson DJ, Rubenfeld GD (2004). Changing pattern of ventilator settings in patients without acute lung injury: changes over 11 years in a single institution. *Chest*.

[18] Bendixen HH, Hedley-whyte J, Laver MB (1963). Impaired oxygenation in surgical patients during general anesthesia with controlled ventilation. *The New England Journal of Medicine*.

[19] Miller RD (2005). *Anesthesia*.

[20] Ashbaugh DG, Bigelow DB, Petty TL, Levine BE (1967). Acute respiratory distress in adults. *Lancet*.

[21] Kumar A, Falke KJ, Geffin B (1970). Continuous positive-pressure ventilation in acute respiratory failure. *New England Journal of Medicine*.

[22] Falke KJ, Pontoppidan H, Kumar A, Leith DE, Geffin B, Laver MB (1972). Ventilation with end-expiratory pressure in acute lung disease. *Journal of Clinical Investigation*.

[23] Suter PM, Fairley B, Isenberg MD (1975). Optimum end-expiratory airway pressure in patients with acute pulmonary failure. *New England Journal of Medicine*.

[24] Mathru M, Rao TLK, Venus B (1983). Ventilator-induced barotrauma in controlled mechanical ventilation versus intermittent mandatory ventilation. *Critical Care Medicine*.

[25] dos Santos CC, Slutsky AS (2000). Invited review: mechanisms of ventilator-induced lung injury: a perspective. *Journal of Applied Physiology*.

[26] Wrigge H, Uhlig U, Zinserling J (2004). The effects of different ventilatory settings on pulmonary and systemic inflammatory responses during major surgery. *Anesthesia and Analgesia*.

[27] Hickling KG (1992). Low volume ventilation with permissive hypercapnia in the Adult Respiratory Distress Syndrome. *Clinical Intensive Care*.

[28] Hickling KG, Walsh J, Henderson S, Jackson R (1994). Low mortality rate in adult respiratory distress syndrome using low-volume, pressure-limited ventilation with permissive hypercapnia: a prospective study. *Critical Care Medicine*.

[29] Meade MO, Cook DJ, Guyatt GH (2008). Ventilation strategy using low tidal volumes, recruitment maneuvers, and high positive end-expiratory pressure for acute lung injury and acute respiratory distress syndrome: a randomized controlled trial. *Journal of the American Medical Association*.

[30] Brower RG, Lanken PN, MacIntyre N (2004). Higher versus lower positive end-expiratory pressures in patients with the acute respiratory distress syndrome. *New England Journal of Medicine*.

[31] Mercat A, Richard J-CM, Vielle B (2008). Positive end-expiratory pressure setting in adults with acute lung injury and acute respiratory distress syndrome: a randomized controlled trial. *Journal of the American Medical Association*.

[32] Villar J, Kacmarek RM, Pérez-Méndez L, Aguirre-Jaime A (2006). A high positive end-expiratory pressure, low tidal volume ventilatory strategy improves outcome in persistent acute respiratory distress syndrome: a randomized, controlled trial. *Critical Care Medicine*.

[33] Dellinger RP, Levy MM, Carlet JM (2008). Surviving Sepsis Campaign: international guidelines for management of severe sepsis and septic shock: 2008. *Critical Care Medicine*.

[34] Briel M, Meade M, Mercat A (2010). Higher vs lower positive end-expiratory pressure in patients with acute lung injury and acute respiratory distress syndrome: systematic review and meta-analysis. *Journal of the American Medical Association*.

[35] Gajic O, Dara SI, Mendez JL (2004). Ventilator-associated lung injury in patients without acute lung injury at the onset of mechanical ventilation. *Critical Care Medicine*.

[36] Gajic O, Frutos-Vivar F, Esteban A, Hubmayr RD, Anzueto A (2005). Ventilator settings as a risk factor for acute respiratory distress syndrome in mechanically ventilated patients. *Intensive Care Medicine*.

[37] Determann RM, Royakkers A, Wolthuis EK (2010). Ventilation with lower tidal volumes as compared with conventional tidal volumes for patients without acute lung injury: a preventive randomized controlled trial. *Critical Care*.

[38] Mascia L, Pasero D, Slutsky AS (2010). Effect of a lung protective strategy for organ donors on eligibility and availability of lungs for transplantation: a randomized controlled trial. *Journal of the American Medical Association*.

[39] http://www.unos.org/docs/Critical_Pathway.pdf.

[40] Shemie SD, Ross H, Pagliarello J (2006). Organ donor management in Canada: recommendations of the forum on Medical Management to Optimize Donor Organ Potential. *Canadian Medical Association Journal*.

[41] Lee PG, Helsmoortel CM, Cohn SM, Fink MP (1990). Are low tidal volumes safe?. *Chest*.

[42] Fernández-Pérez ER, Keegan MT, Brown DR, Hubmayr RD, Gajic O (2006). Intraoperative tidal volume as a risk factor for respiratory failure after pneumonectomy. *Anesthesiology*.

[43] Michelet P, D’Journo XB, Roch A (2006). Protective ventilation influences systemic inflammation after esophagectomy: a randomized controlled study. *Anesthesiology*.

[44] Weingarten TN, Whalen FX, Warner DO (2010). Comparison of two ventilatory strategies in elderly patients undergoing major abdominal surgery. *British Journal of Anaesthesia*.

[45] Fernández-Pérez ER, Sprung J, Afessa B (2009). Intraoperative ventilator settings and acute lung injury after elective surgery: a nested case control study. *Thorax*.

[46] Hughes CG, Weavind L, Banerjee A, Mercaldo ND, Schildcrout JS, Pandharipande PP (2010). Intraoperative risk factors for acute respiratory distress syndrome in critically ill patients. *Anesthesia and Analgesia*.

[47] Kor DJ, Warner DO, Alsara A (2011). Derivation and diagnostic accuracy of the surgical lung injury prediction model. *Anesthesiology*.

[48] Warren OJ, Smith AJ, Alexiou C (2009). The inflammatory response to cardiopulmonary bypass—part 1-mechanisms of pathogenesis. *Journal of Cardiothoracic and Vascular Anesthesia*.

[49] Zupancich E, Paparella D, Turani F (2005). Mechanical ventilation affects inflammatory mediators in patients undergoing cardiopulmonary bypass for cardiac surgery: a randomized clinical trial. *Journal of Thoracic and Cardiovascular Surgery*.

[50] Reis Miranda D, Gommers D, Struijs A (2005). Ventilation according to the open lung concept attenuates pulmonary inflammatory response in cardiac surgery. *European Journal of Cardiothoracic Surgery*.

[51] Koner O, Celebi S, Balci H, Cetin G, Karaoglu K, Cakar N (2004). Effects of protective and conventional mechanical ventilation on pulmonary function and systemic cytokine release after cardiopulmonary bypass. *Intensive Care Medicine*.

[52] Wrigge H, Uhlig U, Baumgarten G (2005). Mechanical ventilation strategies and inflammatory responses to cardiac surgery: a prospective randomized clinical trial. *Intensive Care Medicine*.

[53] Sundar S, Novack V, Jervis K (2011). Influence of low tidal volume ventilation on time to extubation in cardiac surgical patients. *Anesthesiology*.

[54] Lellouche F, Dionne S, Simard S, Bussières J, Dagenais F Traditional and high tidal volumes are associated with prolonged mechanical ventilation and organ failure after cardiac surgery.

[55] Wrigge H, Zinserling J, Stuber F (2000). Effects of mechanical ventilation on release of cytokines into systemic circulation in patients with normal pulmonary function. *Anesthesiology*.

[56] Choi G, Wolthuis EK, Bresser P (2006). Mechanical ventilation with lower tidal volumes and positive end-expiratory pressure prevents alveolar coagulation in patients without lung injury. *Anesthesiology*.

[57] Zanella A, Bellani G, Pesenti A (2010). Airway pressure and flow monitoring. *Current Opinion in Critical Care*.

[58] Leary TS, Milner QJW, Niblett DJ (2000). The accuracy of the estimation of body weight and height in the intensive care unit. *European Journal of Anaesthesiology*.

[59] Richard JC, Maggiore SM, Jonson B, Mancebo J, Lemaire F, Brochard L (2001). Influence of tidal volume on alveolar recruitment: respective role of PEEP and a recruitment maneuver. *American Journal of Respiratory and Critical Care Medicine*.

[60] Déry R, Pelletter J, Jacques A, Clavet M, Houde J (1965). Alveolar collapse induced by denitrogenation. *Canadian Anaesthetists' Society Journal*.

[61] Aboab J, Jonson B, Kouatchet A, Taille S, Niklason L, Brochard L (2006). Effect of inspired oxygen fraction on alveolar derecruitment in acute respiratory distress syndrome. *Intensive Care Medicine*.

[62] Hedenstierna G, Edmark L (2010). Mechanisms of atelectasis in the perioperative period. *Best Practice and Research*.

[63] Jubran A (2004). Pulse oximetry. *Intensive Care Medicine*.

[64] Manzano F, Fernández-Mondéjar E, Colmenero M (2008). Positive-end expiratory pressure reduces incidence of ventilator-associated pneumonia in nonhypoxemic patients. *Critical Care Medicine*.

[65] Metnitz PGH, Metnitz B, Moreno RP (2009). Epidemiology of mechanical ventilation: analysis of the SAPS 3 database. *Intensive Care Medicine*.

[66] Talmor D, Sarge T, Malhotra A (2008). Mechanical ventilation guided by esophageal pressure in acute lung injury. *New England Journal of Medicine*.

[67] O’Croinin D, Ni Chonghaile M, Higgins B, Laffey JG (2005). Bench-to-bedside review: permissive hypercapnia. *Critical Care*.

[68] Lellouche F, Taillé S, Lefrançois F (2009). Humidification performance of 48 passive airway humidifiers comparison with manufacturer data. *Chest*.

[69] Prin S, Chergui K, Augarde R, Page B, Jardin F, Vieillard-Baron A (2002). Ability and safety of a heated humidifier to control hypercapnic acidosis in severe ARDS. *Intensive Care Medicine*.

[70] Prat G, Renault A, Tonnelier JM (2003). Influence of the humidification device during acute respiratory distress syndrome. *Intensive Care Medicine*.

[71] Campbell RS, Davis K, Johannigman JA, Branson RD (2000). The effects of passive humidifier dead space on respiratory variables in paralyzed and spontaneously breathing patients. *Respiratory Care*.

[72] Morán I, Bellapart J, Vari A, Mancebo J (2006). Heat and moisture exchangers and heated humidifiers in acute lung injury/acute respiratory distress syndrome patients. Effects on respiratory mechanics and gas exchange. *Intensive Care Medicine*.

[73] Hinkson CR, Benson MS, Stephens LM, Deem S (2006). The effects of apparatus dead space on PaCO_2_ in patients receiving lung-protective ventilation. *Respiratory Care*.

[74] Richecoeur J, Lu Q, Vieira SR (1999). Expiratory washout versus optimization of mechanical ventilation during permissive hypercapnia in patients with severe acute respiratory distress syndrome. *American Journal of Respiratory and Critical Care Medicine*.

[75] Scales DC, Adhikari NKJ (2008). Lost in (knowledge) translation: “all breakthrough, no follow through”?. *Critical Care Medicine*.

[76] Deans KJ, Minneci PC, Cui X, Banks SM, Natanson C, Eichacker PQ (2005). Mechanical ventilation in ARDS: one size does not fit all. *Critical Care Medicine*.

[77] Gattinoni L (2011). Counterpoint: is low tidal volume mechanical ventilation preferred for all patients on ventilation? No. *Chest*.

[78] Hubmayr RD, Gattinoni L (2011). Point: is low tidal volume mechanical ventilation preferred for all patients on ventilation? Yes. *Chest*.

[79] MacNaughton PD, Evans TW (1994). Measurement of lung volume and DL(CO) in acute respiratory failure. *American Journal of Respiratory and Critical Care Medicine*.

[80] Rylander C, Tylén U, Rossi-Norrlund R, Herrmann P, Quintel M, Bake B (2005). Uneven distribution of ventilation in acute respiratory distress syndrome. *Critical Care*.

[81] Chiumello D, Carlesso E, Cadringher P (2008). Lung stress and strain during mechanical ventilation for acute respiratory distress syndrome. *American Journal of Respiratory and Critical Care Medicine*.

[82] Gattinoni L, Carlesso E, Caironi P (2012). Stress and strain within the lung. *Current Opinion in Critical Care*.

[83] Ferguson ND, Frutos-Vivar F, Esteban A (2005). Acute respiratory distress syndrome: underrecognition by clinicians and diagnostic accuracy of three clinical definitions. *Critical Care Medicine*.

[84] Walkey AJ, Wiener RS Risk factors for underuse of lung-protective ventilation in acute lung injury.

[85] Krieger BP (2009). Hyperinflation and intrinsic positive end-expiratory pressure: less room to breathe. *Respiration*.

[86] Richard JC, Brochard L, Breton L (2002). Influence of respiratory rate on gas trapping during low volume ventilation of patients with acute lung injury. *Intensive Care Medicine*.

[87] Brochard L (2002). Intrinsic (or auto-) PEEP during controlled mechanical ventilation. *Intensive Care Medicine*.

[88] Blanch L, Bernabé F, Lucangelo U (2005). Measurement of air trapping, intrinsic positive end-expiratory pressure, and dynamic hyperinflation in mechanically ventilated patient. *Respiratory Care*.

[89] Unroe M, MacIntyre N (2010). Evolving approaches to assessing and monitoring patient-ventilator interactions. *Current Opinion in Critical Care*.

[90] Schmidt M, Demoule A, Polito A (2011). Dyspnea in mechanically ventilated critically ill patients. *Critical Care Medicine*.

[91] Kallet RH, Campbell AR, Dicker RA, Katz JA, Mackersie RC (2006). Effects of tidal volume on work of breathing during lung-protective ventilation in patients with acute lung injury and acute respiratory distress syndrome. *Critical Care Medicine*.

[92] Hess DR, Thompson BT (2006). Patient-ventilator dyssynchrony during lung protective ventilation: what’s a clinician to do?. *Critical Care Medicine*.

[93] Nilsestuen JO, Hargett KD (2005). Using ventilator graphics to identify patient-ventilator asynchrony. *Respiratory Care*.

[94] Kahn JM, Andersson L, Karir V, Polissar NL, Neff MJ, Rubenfeld GD (2005). Low tidal volume ventilation does not increase sedation use in patients with acute lung injury. *Critical Care Medicine*.

[95] Papazian L, Forel J-M, Gacouin A (2010). Neuromuscular blockers in early acute respiratory distress syndrome. *New England Journal of Medicine*.

[96] Rubenfeld GD, Cooper C, Carter G, Thompson BT, Hudson LD (2004). Barriers to providing lung-protective ventilation to patients with acute lung injury. *Critical Care Medicine*.

[97] Fuchs H, Mendler MR, Scharnbeck D, Ebsen M, Hummler HD (2011). Very low tidal volume ventilation with associated hypercapnia—effects on lung injury in a model for acute respiratory distress syndrome. *PLoS ONE*.

[98] de Graaff AE, Dongelmans DA, Binnekade JM, de Jonge E (2011). Clinicians’ response to hyperoxia in ventilated patients in a Dutch ICU depends on the level of FiO2. *Intensive Care Medicine*.

[99] Altemeier WA, Sinclair SE (2007). Hyperoxia in the intensive care unit: why more is not always better. *Current Opinion in Critical Care*.

[100] Floyd TF, Clark JM, Gelfand R (2003). Independent cerebral vasoconstrictive effects of hyperoxia and accompanying arterial hypocapnia at 1 ATA. *Journal of Applied Physiology*.

[101] Farquhar H, Weatherall M, Wijesinghe M (2009). Systematic review of studies of the effect of hyperoxia on coronary blood flow. *American Heart Journal*.

[102] Wijesinghe M, Perrin K, Ranchord A, Simmonds M, Weatherall M, Beasley R (2009). Routine use of oxygen in the treatment of myocardial infarction: systematic review. *Heart*.

[103] Graham ID, Logan J, Harrison MB (2006). Lost in knowledge translation: time for a map?. *Journal of Continuing Education in the Health Professions*.

[104] Weinert CR, Gross CR, Marinelli WA (2003). Impact of randomized trial results on acute lung injury ventilator therapy in teaching hospitals. *American Journal of Respiratory and Critical Care Medicine*.

[105] Young MP, Manning HL, Wilson DL (2004). Ventilation of patients with acute lung injury and acute respiratory distress syndrome: has new evidence changed clinical practice?. *Critical Care Medicine*.

[106] Umoh NJ, Fan E, Mendez-Tellez PA (2008). Patient and intensive care unit organizational factors associated with low tidal volume ventilation in acute lung injury. *Critical Care Medicine*.

[107] Checkley W, Brower R, Korpak A, Thompson BT (2008). Effects of a clinical trial on mechanical ventilation practices in patients with acute lung injury. *American Journal of Respiratory and Critical Care Medicine*.

[108] Esteban A, Ferguson ND, Meade MO (2008). Evolution of mechanical ventilation in response to clinical research. *American Journal of Respiratory and Critical Care Medicine*.

[109] Bloomfield R, Steel E, MacLennan G, Noble DW (2006). Accuracy of weight and height estimation in an intensive care unit: implications for clinical practice and research. *Critical Care Medicine*.

[110] Hendershot KM, Robinson L, Roland J, Vaziri K, Rizzo AG, Fakhry SM (2006). Estimated height, weight, and body mass index: implications for research and patient safety. *Journal of the American College of Surgeons*.

[111] Maskin LP, Attie S, Setten M (2010). Accuracy of weight and height estimation in an intensive care unit. *Anaesthesia and Intensive Care*.

[112] Han S, Martin GS, Maloney JP (2011). Short women with severe sepsis-related acute lung injury receive lung protective ventilation less frequently: an observational cohort study. *Critical Care*.

[113] iAnthropometer ICU1 http://itunes.apple.com/ca/app/ianthropometer-icu-1/id428778012?mt=8.

[114] Bojmehrani A, Bouchard C, Bouchard PA, L'Her E, Lellouche F (2011). Evaluation of new tool to measure patient's height during mechanical ventilation: impact on protective ventilation (freesize). *Respiratory Care Open Forum*.

[115] Lellouche F, Bouchard PA, Wysocki M (2010). Prospective randomized controlled study comparing conventional ventilation versus a fully closed-loop ventilation (IntelliVent) in post cardiac surgery ICU patients. *American Journal of Respiratory and Critical Care Medicine*.

[116] Iotti GA, Polito A, Belliato M (2010). Adaptive support ventilation versus conventional ventilation for total ventilatory support in acute respiratory failure. *Intensive Care Medicine*.

[117] Gruber PC, Gomersall CD, Leung P (2008). Randomized controlled trial comparing adaptive-support ventilation with pressure-regulated volume-controlled ventilation with automode in weaning patients after cardiac surgery. *Anesthesiology*.

[118] Burns KEA, Lellouche F, Lessard MR (2008). Automating the weaning process with advanced closed-loop systems. *Intensive Care Medicine*.

[119] Branson RD, Davis K (2008). Does closed loop control of assist control ventilation reduce ventilator-induced lung injury?. *Clinics in Chest Medicine*.

[120] Schultz MJ, Haitsma JJ, Slutsky AS, Gajic O (2007). What tidal volumes should be used in patients without acute lung injury?. *Anesthesiology*.

